# A novel composite formulation of palmitoylethanolamide and quercetin decreases inflammation and relieves pain in inflammatory and osteoarthritic pain models

**DOI:** 10.1186/s12917-017-1151-z

**Published:** 2017-08-02

**Authors:** Domenico Britti, Rosalia Crupi, Daniela Impellizzeri, Enrico Gugliandolo, Roberta Fusco, Carlo Schievano, Valeria Maria Morittu, Maurizio Evangelista, Rosanna Di Paola, Salvatore Cuzzocrea

**Affiliations:** 1 Department of Health Sciences V. le Europa, Campus S. Venuta, Germaneto, 88100 Catanzaro, Italy; 2 Department of Chemical, Biological, Pharmaceutical and Environmental Sciences, University of Messina, Viale Ferdinando Stagno D’Alcontres 31-, I-98166 Messina, Italy; 3 Innovative Statistical Research SRL, Prato Della Valle 24, I-35123 Padova, Italy; 4 Institute of Anaesthesiology and Reanimation, Catholic University of Sacred Heart, Rome, Italy; 5 Manchester Biomedical Research Centre, Manchester Royal Infirmary, School of Medicine, University of Manchester, Manchester, UK

**Keywords:** Osteoarthritis, Disease models, Pain, Inflammation, Drug combinations, Palmitoylethanolamide, Quercetin, Co-ultra micronization, N-acylethanolamines

## Abstract

**Background:**

Osteoarthritis (OA) is a common progressive joint disease in dogs and cats. The goal of OA treatment is to reduce inflammation, minimize pain, and maintain joint function. Currently, non-steroidal anti-inflammatory drugs (e.g., meloxicam) are the cornerstone of treatment for OA pain, but side effects with long-term use pose important challenges to veterinary practitioners when dealing with OA pain. Palmitoylethanolamide (PEA) is a naturally-occurring fatty acid amide, locally produced on demand by tissues in response to stress. PEA endogenous levels change during inflammatory and painful conditions, including OA, i.e., they are typically increased during acute conditions and decreased in chronic inflammation. Systemic treatment with PEA has anti-inflammatory and pain-relieving effects in several disorders, yet data are lacking in OA. Here we tested a new composite, i.e., PEA co-ultramicronized with the natural antioxidant quercetin (PEA-Q), administered orally in two different rat models of inflammatory and OA pain, namely carrageenan paw oedema and sodium monoiodoacetate (MIA)-induced OA. Oral treatment with meloxicam was used as benchmark.

**Results:**

PEA-Q decreased inflammatory and hyperalgesic responses induced by carrageenan injection, as shown by: (i) paw oedema reduction, (ii) decreased severity in histological inflammatory score, (iii) reduced activity of myeloperoxidase, i.e., a marker of inflammatory cell infiltration, and (iv) decreased thermal hyperalgesia. Overall PEA-Q showed superior effects compared to meloxicam. In MIA-treated animals, PEA-Q exerted the following effects: (i) reduced mechanical allodynia and improved locomotor function, (ii) protected cartilage against MIA-induced histological damage, and (iii) counteracted the increased serum concentration of tumor necrosis factor alpha, interleukin 1 beta, metalloproteases 1, 3, 9 and nerve growth factor. The magnitude of these effects was comparable to, or even greater than, those of meloxicam.

**Conclusion:**

The present findings shed new light on some of the inflammatory and nociceptive pathways and mediators targeted by PEA-Q and confirm its anti-inflammatory and pain-relieving effects in rodent OA pain models. The translatability of these observations to canine and feline OA pain is currently under investigation.

## Background

Osteoarthritis (OA) represents one of the most frequently occurring painful conditions in both humans and small animals [1]. In dogs, it affects approximately 20% of those over the age of 1 year [2]. OA is also very common in elderly domesticated cats, its prevalence being greater than 50% [3]. Clinical signs (i.e., lameness, stiffness, behavioral and lifestyle changes) are largely related to persistent and chronic pain [1–3], i.e. a mixed phenomenon involving both inflammatory and neuropathic mechanisms at the peripheral (joint) and central (spinal and supraspinal) levels [4]. Currently, the most frequently used analgesics for canine and feline OA are non-steroidal anti-inflammatory drugs (NSAIDs) [4]. Despite their widespread use, the main drawbacks of NSAIDs relate to both poor efficacy against the neuropathic component of OA pain [5] and unwanted side effects, especially with long-term use [6]. Thus, research is now focused on the identification of more effective and safe analgesic tools, as part of an ideal multimodal management of pain in veterinary OA patients [4, 7].

Given the aetiological and clinical heterogeneity of canine and feline OA, several animal models have been employed to investigate the multifaceted mechanisms of OA pain and to evaluate the analgesic efficacy of different compounds [8, 9]. Experimental OA pain models that provide ease of induction and reproducibility without the need for surgery are favored [9]. Of these, subplantar carrageenan (CAR) injection is recognized as a model of acute and highly reproducible inflammatory pain [10, 11], the main signs of inflammation - oedema, hyperalgesia, and erythema – developing immediately following subcutaneous injection. Neutrophils readily migrate to sites of CAR-induced inflammation and generate pro-inflammatory mediators, such as bradykinin, histamine, and reactive oxygen and nitrogen species, with consequent sensitization of local nociceptors and inflammatory pain development [11]. Recently, the intra-articular injection of sodium monoiodoacetate (MIA) has been suggested to be more predictive of OA pain drug efficacy than other models [8, 9]. MIA inhibits a key glycolytic enzyme, leading to chondrocyte cell death and bone lesions [9]. Pain-related characteristics in the MIA model are considered to originate from an inflammatory pain state induced by the local increase of inflammatory cytokines, followed by gradual initiation of neuronal cell injury and nerve sensitization, culminating in neuropathic pain [9, 12].

Palmitoylethanolamide (PEA) is a naturally-occurring N-acylethanolamine and a congener of the endocannabinoid anandamide [13]. First discovered in the 1950s as the active anti-inflammatory component of some food sources (recently reviewed in [14]), PEA has emerged as a disease-modifying agent in conditions eliciting both inflammatory and neuropathic pain, both in experimental (e.g. intraplantar injection of formalin, chronic constriction injury, viscerovisceral hyperalgesia) and naturally occurring conditions (eg. diabetic neuropathy, low back pain and pelvic pain in human patients) [13–15]. Moreover, PEA is currently considered a negative regulator of tissue inflammation [14, 16]. However, its effect on joint disease-related pain remains to be fully investigated. Available data are mainly limited to changes in endogenous levels of PEA, with an increase being reported in the spinal cord of MIA-treated rats [17] and a marked decrease in the synovial fluid of human patients with OA or rheumatoid arthritis [18]. These findings posit that PEA metabolism is deregulated during joint disorders and that exogenous supply of PEA may be beneficial in such conditions [19].

Oxidative stress is considered to be an important etiologic factor in OA [20], and the antioxidant quercetin has been used with success as an adjunct in human and experimental arthritic diseases [21, 22]. New formulations of PEA have recently been introduced in the Italian and international health market, both in the human and veterinary fields [14]. These mainly comprise micronized or ultramicronized formulations of PEA (i.e., micron- or submicron-sized particles with better solubility), that show superior activity compared to naïve PEA [23]. In some formulations, PEA is micronized (or ultramicronized) together with natural polyphenols, showing a synergistic effect and the ability to target also the oxidative stress cascade [14]. The newest of these formulations is a co-ultramicronized composite of PEA with quercetin (PEA-Q).

Based on the above considerations, the present study aimed to investigate the anti-inflammatory and pain-relieving effects of PEA-Q. Two sets of experiments were carried out. In the first (preliminary study) the CAR-induced inflammatory rat pain model was used. The encouraging results thus obtained led us to then test PEA-Q in the MIA-induced rat OA pain model. In both sets of experiments pain thresholds were evaluated and inflammatory/nociceptive markers measured. In all these studies meloxicam was used as a representative NSAID commonly prescribed in Europe for treating chronic OA pain in dogs and cats.

## Methods

### Animals

This study was performed on Sprague–Dawley male rats (200–230 g, 7 weeks old, Envigo RMS Srl, S. Pietro al Natisone, Udine, Italy). Ten rats were used for each treatment group (see below). Food and water were available ad libitum. The University of Messina Review Board for the care and use of animals authorized the study. Animal care was in accordance with Italian regulations on protection of animals used for experimental and other scientific purposes (D.M.116192) as well as with EEC regulations (O.J. of E.C. L 358/1 12/18/1986).

### Reagents

Co-ultramicronized PEA-Q was kindly provided by Epitech group SpA (Saccolongo, Italy). PEA-Q is the result of the joint ultramicronization - by jet-milling technology - of a mixture made of PEA and quercetin in a 5:1 ratio by mass. All other compounds were obtained from Sigma-Aldrich, Milan, Italy. All chemicals were of the highest commercial grade available. All stock solutions were prepared in non-pyrogenic saline (0.9% NaCl, Baxter International, Rome, Italy).

### Experimental models

#### CAR-induced inflammation

Rats were anesthetized with 5.0% isoflurane in 100% O_2_ (Baxter International, Rome, Italy) and received a subplantar injection of CAR (0.1 ml/rat of a 1% suspension in saline) (Sigma–Aldrich, Milan, Italy) as previously reported [11]. Injections were performed using a 27-gauge needle inserted into the pad region of the glabrous skin on the underside of the right hind paw. At 6 h following CAR injection, rats were sacrificed by anaesthetic (isoflurane) overdose.

All analyses conducted in CAR-injected rats (see details below) were performed in a blinded manner.

#### MIA induction model

OA was induced by intra-articular injection of MIA in the right knee joint as previously described [24]. On day 0, rats were anesthetized with 5.0% isoflurane in 100% O_2_. A volume of 25 μl saline +3 mg of MIA was injected into the knee joint through the right intrapatellar ligament. The left knee received an equal volume of saline. MIA was prepared in sterile conditions and injected using a 50 μl Hamilton syringe with a 27 gauge needle that was inserted into the joint for about 2–3 mm. On day 21 post-MIA injection, rats were sacrificed by anaesthetic overdose and perfused with 4% paraformaldehyde. All analyses conducted in MIA-injected rats (see details below) were performed in a blinded manner.

### Experimental design

#### Treatment groups

The experiment was divided in two steps. First, we conducted a preliminary study to evaluate the effect of PEA-Q on inflammation and pain-related events (i.e., thermal hyperalgesia) in the CAR-induced paw oedema model. Rats were arbitrarily allocated to the following treatment groups, each compound being administered orally 30 min before CAR injection:

(i) CAR + vehicle (saline): rats were subjected to CAR-induced paw oedema, as described above (*N* = 10);
(ii) CAR *+* quercetin: same as the CAR + vehicle group plus 3.3 mg/kg quercetin dissolved in carboxymethylcellulose (1.5% *w*/*v* in saline) (*N* = 10);
(iii) CAR + PEA-Q (10): same as the CAR + vehicle group plus 10 mg/kg PEA-Q dissolved in carboxymethylcellulose (1.5% *w*/*v* in saline) (*N* = 10);
(iv) CAR + PEA-Q (20): same as the CAR + vehicle group plus 20 mg/kg PEA-Q dissolved in carboxymethylcellulose (1.5% *w*/*v* in saline) (*N* = 10);
(v) CAR + meloxicam: same as the CAR + vehicle group plus 0.2 mg/kg meloxicam dissolved in saline (*N* = 10).
(vi) sham group: same surgical procedure as the CAR group, except that saline was administered instead of CAR (*N* = 10).

Doses were chosen based on a dose-response studied carried out in our lab and on existing literature data. The dose of quercetin used is equivalent to 20 mg/kg PEA-Q. Based on preliminary results, we evaluated the anti-inflammatory and analgesic effects of PEA-Q in an experimental model of MIA-induced OA pain (definitive study). Rats were randomly divided into the following treatment groups, each compound being administered orally three times per week for 21 days, starting the third day after MIA injection:

(i) MIA + vehicle (saline): rats were subjected to induction of OA pain as described above (*N* = 10).
(ii) MIA + PEA-Q (10): same as the MIA + vehicle group plus 10 mg/kg PEA-Q dissolved in carboxymethylcellulose (1.5% *w*/*v* in saline) (*N* = 10);
(iii) MIA + PEA-Q (20): same as the MIA + vehicle group plus 20 mg/kg PEA-Q dissolved in carboxymethylcellulose (1.5% *w*/*v* in saline) (*N* = 10);
(iv) MIA + meloxicam: same as the MIA + vehicle group plus 0.2 mg/kg meloxicam dissolved in carboxymethylcellulose (1.5% *w*/*v* in saline) (*N* = 10);
(v) sham-operated group: rats received an intra-articular injection of saline (25 μl) instead of MIA (*N* = 10).

Quercetin was not tested in this set of experiments, given the lack of effect observed in the previous set. Doses were chosen based on a dose-response study carried out in our lab and on existing literature data. The timing of oral administration was based on a previous study [25].

The effect of PEA alone was not investigated, since oral administration of micronized and ultramicronized PEA had been shown to reduce inflammation and pain in several experimental models, the effective dose being 10 mg/kg [13, 14], thus higher than the dose equivalent to 10 mg/kg PEA-Q used here. Moreover, oral administration of naïve PEA (non-micronized) did not exert any significant effect against inflammation and hyperalgesia up to 10 mg/kg [23].

### Assessment of CAR-induced paw oedema

Oedma was assessed by directly measuring changes in paw volume using a plethysmometer (Ugo Basile, Varese, Italy), as previously described [11]. Paw volume was measured immediately prior to CAR injection and thereafter at hourly intervals for 6 h. Oedema was expressed as the increase in paw volume (ml) after CAR injection relative to pre-injection value. Six hours after CAR injection, percentage inhibition (protection) against oedema formation was calculated as follows and taken as an index of anti-inflammatory activity:

Percentage inhibition of inflammation = [(Vc-Vt)/Vc] × 100, where;

Vc = mean paw oedema volume in the control group at 6 h;

Vt = mean paw oedema volume in the drug-treated group at 6 h.

### Pain-related behavioral analysis in the CAR model

The hyperalgesic response to heat was measured at different time points (0, 30 min, 1, 2, 3, 4 and 5 h) based on the method described by Hargreaves et al. [26] using Plantar Test 7371 (Ugo Basile, Italy). Briefly, animals were allowed to move freely within an open-topped transparent plastic chamber. After an acclimation period, a mobile infrared (I.R.) radiant heating source (IR 60) was placed under the glass floor and focused onto the hind paw. When the animal felt pain and withdrew its paw, the I.R. source switched off and the reaction time counter stopped. The withdrawal latency to the nearest 0.1 s was then automatically determined and recorded. A cutoff time of 20 s was set, i.e., if the rat failed to respond by 20 s the test was stopped in order to prevent tissue damage in non-responsive animals. Each point represents the delta change (sec) in withdrawal latency (withdrawal latency of contralateral minus withdrawal latency of injected paw) at each time point. Results are expressed as paw withdrawal latency changes (sec).

Percentage anti-hyperalgesic activity 5 h after CAR injection was calculated as follows.

Percentage anti-hyperalgesic activity = [(Vt-Vc)/Vt] × 100 where:

Vc = mean paw withdrawal latency in the control group at 5 h;

Vt = mean paw withdrawal latency in the drug-treated group at 5 h.

### Histological analysis following CAR injection

Six hours after intraplantar CAR injection, animals were terminally anesthetized and paw biopsies collected. Histological analysis of haematoxylin and eosin-stained paw tissue was performed as previously described [23]. The degree of paw damage was evaluated according to Bang and Coll. [27], on a six-point score: 0 = no inflammation, 1 = mild inflammation, 2 = mild/moderate inflammation, 3 = moderate inflammation, 4 = moderate/severe inflammation and 5 = severe inflammation. The photographs obtained (*n* = 5 photos from five slides for each sample) were collected from all animals in each experimental group. The histological coloration (5 slides for each same sample) was repeated three times on different days.

### Myeloperoxidase (MPO) activity following CAR injection

MPO activity, an index of neutrophilic granulocyte infiltration, was evaluated as previously described [28]. Briefly, after animals were terminally anesthetized paw tissues were collected and homogenized in a solution containing 0.5% hexadecyltrimethylammoniumbromide dissolved in 10 mM potassium phosphate buffer (pH 7) and centrifuged for 30 min at 20000 *g* (4 °C). The supernatant was allowed to react with a solution of tetramethylbenzidine (1.6 mM) and 0.1 mM H_2_O_2_. The rate of change in absorbance was measured spectrophotometrically at 650 nm. MPO activity was measured as the quantity of enzyme degrading 1 mM of peroxide min^−1^ at 37 °C, and expressed in units per gram of wet tissue weight.

### Assessment of MIA-induced mechanical allodynia

Mechanical allodynia was evaluated using a dynamic plantar Von Frey hair aesthesiometer on day 0 and 3, 7, 14 and 21 days post-injection (Ugo Basile, Comerio, Italy). Rats were located on a metal mesh surface in a chamber in a room with controlled temperature (21-22^o^ C) and were allowed to adapt for 15 min before testing began. The touch stimulator part was positioned under the rat. When the aesthesiometer was activated, a plastic monofilament touched the paw in the metatarsal region. The filament exercised a gradually increasing force on the plantar surface, starting below the threshold of detection and increasing until the stimulus became painful and the rat removed its paw. The force required to produce a paw withdrawal reflex was recorded automatically and measured in grams. A maximum force of 50 g and a ramp speed of 20 s were used for all the aesthesiometry tests. Paw withdrawal latency (PWL) and paw withdrawal threshold (PWT) were calculated.

### Motor function analysis (walking track analysis) following MIA injection

Motor functional recovery of the rear limb was evaluated by walking track analysis, a reliable and easily quantifiable noninvasive method based on gait analysis by means of specific footprint parameters [29]. In brief, rats were previously trained to walk down a track with a dark end, covered with strips of white paper. Tracks were obtained by wetting the rat’s hind feet with water soluble black ink. Walking track analysis was performed in all animal groups before MIA injection (day 0) and 3, 7, 14 and 21 days post-injection. From the footprints, several measurements are taken between different anatomic landmarks (e.g., width and length of the footprint) and then incorporated in a mathematical formula, allowing the calculation of the functionality index of the sciatic nerve (SFI), with values close to 0 indicating normal function, and values tending to −100 indicating total impairment [29].

### Histological analysis of MIA-injected rats

On day 21 post-MIA injection, rats were sacrificed and perfused with 4% paraformaldehyde. The MIA- and vehicle-injected tibiofemoral joints were dissected and post-fixed in neutral buffered formalin (containing 4% formaldehyde), decalcified in EDTA and processed as previously described. After decalcification, the specimens were embedded in paraffin. Mid-coronal tissue sections (5 μm) were stained for evaluation; all histomorphometric analyses were performed by an observer blinded to the treatment group. Sections were stained with haematoxylin and eosin and observed by light microscopy (Dialux 22 Leitz; Leica Microsystems SpA, Milan, Italy). Histopathological analysis of the cartilage was assessed by the modified Mankin score [30]. Briefly, the score assessed: (i) structure, (ii) cellular abnormalities, and (iii) matrix staining of cartilage sections and ranged from 0 (= normal histology) to 12 (= complete disorganization and hypocellularity). The photographs obtained (*n* = 5 photos from five slides for each sample) were collected from all animals in each experimental group. The histological coloration (5 slides for each same sample) was repeated three times on different days.

### Serum concentration of inflammatory, nociceptive and matrix degradation markers following MIA injection

The concentration of tumor necrosis factor alpha (TNF-α), interleukin-1beta (IL-1β), nerve growth factor (NGF), and matrix metalloproteinase-1-3-9 (MMP-1, MMP-3, MMP-9) were measured in serum using commercial colorimetric ELISA kits (TNF-α, IL-1β, NGF: Thermo Fisher Scientific**,** DBA s.r.l. Milan Italy; MMP-1 MMP-3 MMP-9: Cusabio, DBA s.r.l. Milan Italy).

### Data analysis

Data are expressed as mean ± standard error of the mean (SEM) of *N* observations, *N* representing the number of animals analyzed, with the exception of the ordinal level variable (i.e., histological score), for which median and range were used. In experiments involving histology, the figures are representative of at least three experiments performed on different days. The response over time was analyzed using a generalized linear mixed model (GLMM) for repeated measures, followed by Tukey-Kramer *post-hoc* analysis. One time evaluations with continuous level data were analyzed using ANOVA followed by Bonferroni-Holm post hoc analysis. Kruskal-Wallis test followed by Dunn’s test for *post-hoc* comparisons with Bonferroni-Holm p correction was used for the histological score, due the ordinal level nature of the variable (i.e., 0 to 5 point scale). Data were analyzed using SAS v9.2 (SAS Institute, Cary, NC, USA). The significance threshold was set at 0.05. Exact *p* values are reported, unless less than 1 out of 10,000 (reported as *p* < 0.0001), 0.0001 being the lower limit for the statistical program.

## Results

### Preliminary study

#### A. Effect of PEA-Q on the time-course of CAR-induced paw oedema

**Fig. 1:**
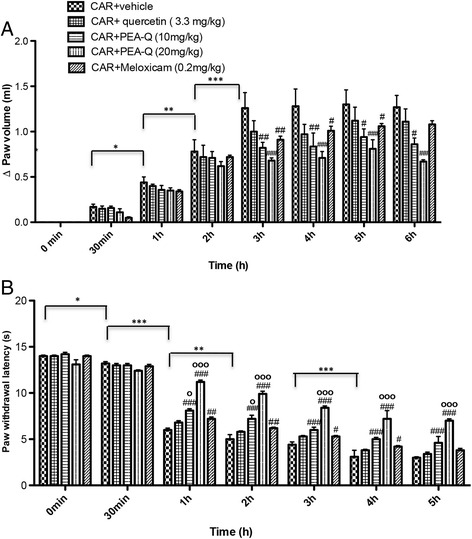
Effects of PEA-Q on CAR-induced paw oedema and heat hyperalgesia. Paw oedema and hyperalgesia were measured at the time points indicated. Oral treatment with PEA-Q (10 and 20 mg/kg) produced a significant improvement in paw volume that, unlike meloxicam, lasted up to the latest time point (**a**). In addition, PEA-Q was more effective in decreasing the hyperalgesia compared meloxicam (**b**). See Methods for further details. Values are means ± SEM of 10 animals for each group. *p < 0.05, **p < 0.001 and ***p < 0.0001 vs previous time point. #*p* < 0.05, ##*p* < 0.001, ###*p* < 0.0001 vs CAR + vehicle. ^°^ *p* < 0.05 and ^°°°^ *p* < 0.0001 vs CAR + meloxicam. The exact *p* values are reported in the text

**Fig. 2:**
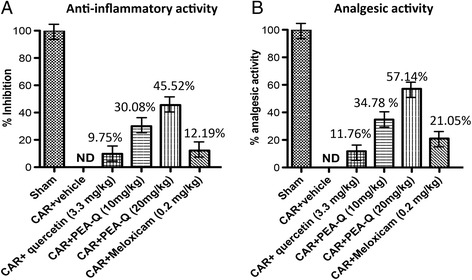
Effects of PEA-Q on CAR-induced inflammation and analgesic activity. Percentages of anti-inflammatory (**a**) and analgesic activity (**b**) for the tested compounds as measured, respectively, at 6 h and 5 h post-CAR injection. See Methods for further details

Intraplantar injection of CAR in rats led to a significant time-dependent increase in paw volume, reaching a peak after 3 h (*p* < 0.0001) (Fig. 1a). Oral treatment with PEA-Q at 10 mg/kg significantly reduced paw volume at 3, 4, 5 and 6 h after injection (*p* = 0.0007, *p* = 0.0005, *p* = 0.0250 and *p* = 0.0029 respectively). The same results were observed for 20 mg/kg (*p* < 0.0001 at all time points) (Fig. 1a). Overall the effect of both doses of PEA-Q was longer compared to meloxicam, whose effect was no more statistically significant at 6 h. No significant difference was observed at any time points between the two doses of PEA-Q or between either dose of PEA-Q and meloxicam. Quercetin did not achieve a significant reduction at any of these time points (Fig. 1a). The percentage of anti-inflammatory activity of the tested compounds (quercetin, 10 and 20 mg/kg PEA-Q, and meloxicam) was, respectively, 10%, 30%, 45% and 12% (Fig. 2a).

#### B. Effect of PEA-Q on the time-course of CAR-induced thermal hyperalgesia

Intraplantar injection of CAR led to a statistically significant time-dependent development of thermal hyperalgesia, latency being increasingly decreased at 30 min, 1 h, 2 h and 4 h after injection (*p* = 0.0209, *p* < 0.0001, *p* = 0.0004, *p* < 0.0001 respectively) (Fig. 1b). Oral treatment with PEA-Q (10 and 20 mg/kg) was efficacious in decreasing the hyperalgesic response from the first to the fifth hour after injection (*p* < 0.0001 for both doses and all time points) (Fig. 1b). Moreover, the higher dose of PEA-Q resulted to be superior to the lower (*p* < 0.0001 at all time points). Similarly to paw oedema, the effect of meloxicam was shorter, statistical significance being lost at the fifth hour. Limited to the first and the second hour post-injection, the anti-hyperalgesic effect exerted by 10 mg/kg PEA-Q was significantly higher than that of meloxicam (*p* = 0.0309 and *p* = 0.0230 respectively). Superiority of 20 mg/kg PEA-Q over meloxicam was maintained at all time points (*p* < 0.0001). Quercetin was unable to change withdrawal latency (Fig. 1b). The percentage of anti-hyperalgesic activity of the tested compounds ranged from 12% (quercetin) to 57% (20 mg/kg PEA-Q) (Fig. 2b).

#### C. Effect of PEA-Q on CAR-induced histological damage

**Fig. 3:**
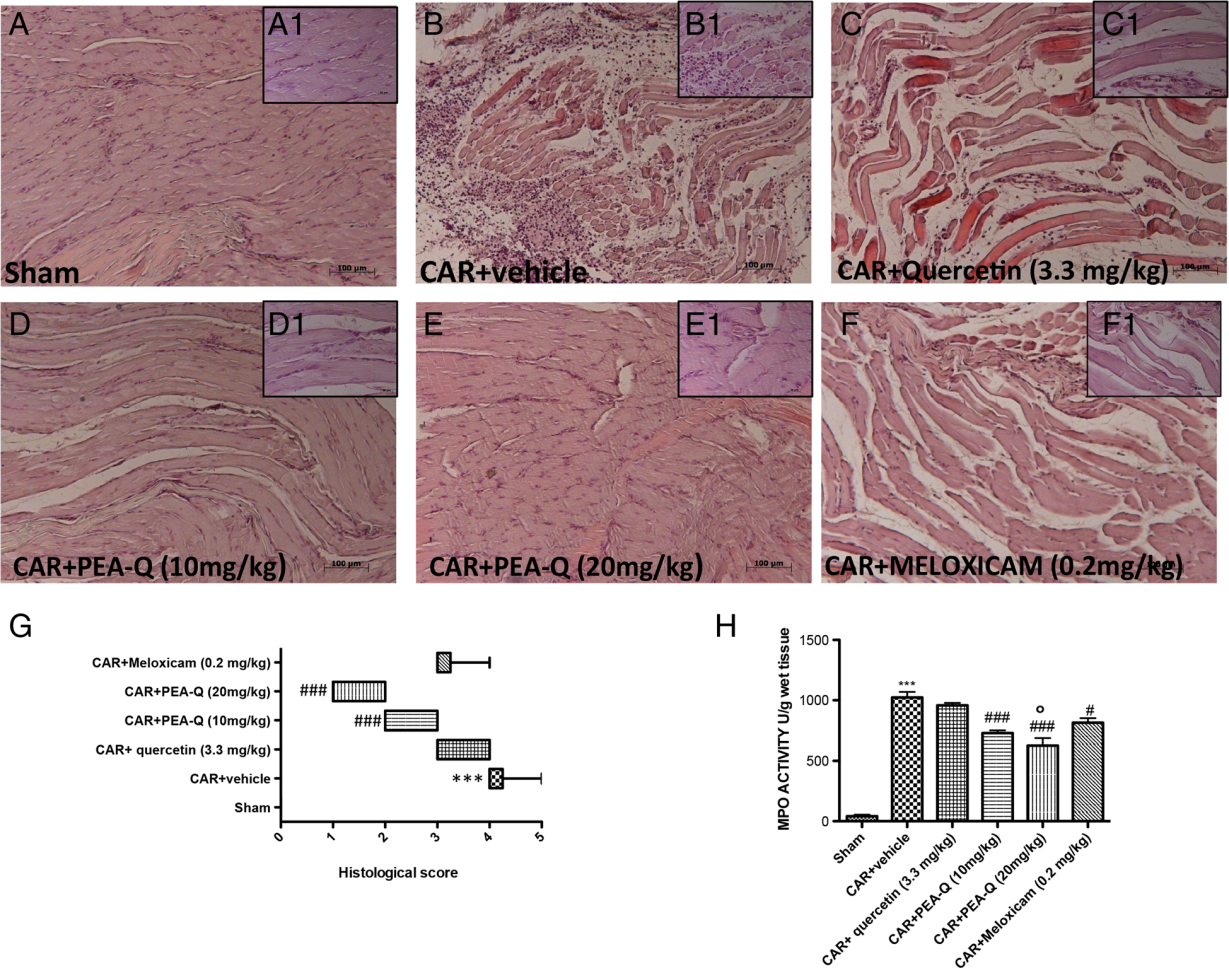
Effects of PEA-Q on CAR-induced histological damage and neutrophil infiltration. Histological evaluation was performed by haematoxylin and eosin staining. Panels **a**-**a1**, sham; panels B-B1, CAR-injected; Panels **c**-**c1**, quercetin treatment; Panels **d**, **d1** and **e**, **e1**, PEA-Q treatment; Panels **f**, **f1** meloxicam treatment. Figures are representative of all animals in each group. Panel **g**, histological score for the various treatment groups. Distribution of data is represented by box plot analysis. Panel **h**, MPO activity. Values are means ± SEM of 10 animals for each group. ***p < 0.0001 vs sham. ^#^ *p* < 0.05 and ^###^ *p* < 0.0001 vs CAR + vehicle. °*p* < 0.05 vs CAR + meloxicam. The exact *p* values are reported in the text

Sham rats exhibited no histological damage (Fig. 3a and inset a1). In contrast, CAR paw injection led to a marked infiltration of inflammatory cells (Fig. 3b and inset b1). Clear improvements were seen in paw tissues of rats treated with PEA-Q at both doses (Fig. 3 d, d1; e, e1), while little effect was observed in the quercetin- and meloxicam-treated groups (Fig. 3c, c1, and Fig. 3f, f1). Histological score in the vehicle group (median 4, range 4–5) was significantly improved by PEA-Q both at 10 mg/kg (median 2, range 2–3) and 20 mg/kg dose (median 1, range 1–2) (*p* < 0.0001 for both doses). Quercetin (median 4, range 3–4) and meloxicam (median 3, range 3–4) did not significantly change the severity of the histological score compared to vehicle (Fig. 3g).

#### D. Effect of PEA-Q on CAR-induced increase in MPO activity

The development of histological damage was associated with a statistically significant increase in MPO activity (*p* < 0.0001), a marker of neutrophilic infiltration (Fig. 3h). Treatment with PEA-Q (*p* < 0.0001, both doses) and meloxicam (*p* = 0.0014), but not quercetin, significantly reduced MPO activity compared to vehicle (Fig. 3h). PEA-Q at the higher dose showed a statistically significant higher effect compared to meloxicam (*p* = 0.0051).

### Definitive study

#### A. Effect of PEA-Q on pain and motor function deficits following MIA induction

**Fig. 4:**
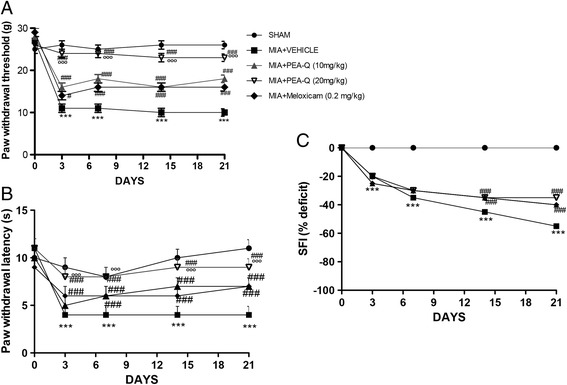
Effects of PEA-Q on MIA-induced OA pain and motor function. Paw withdrawal threshold **a**, paw withdrawal latency **b** and motor function **c** as recorded for each treatment group before and after 3, 7, 14 and 21 days from the intra-articular injection of MIA. Data are means ± SEM of 10 rats for each group. ***p < 0.0001 versus sham. ^#^ *p* < 0.05 and ^###^ *p* < 0.0001 vs MIA + vehicle. °°°*p* < 0.0001 vs MIA + PEA-Q (10 mg/kg) and MIA + meloxicam. The exact *p* values are reported in the text

Because pain is the hallmark of MIA-induced OA, mechanical allodynia in MIA-injected rats was assessed. In the von Frey hair assessment test, PWT (Fig. 4a) and PWL (Fig. 4b) were significantly decreased in the MIA + vehicle group compared to sham animals (*p* < 0.0001 for both variables at any time point). Treatment with PEA-Q at both doses (*p* < 0.0001 at any time point and either dose) and meloxicam (*p* = 0.0103 day 3 and *p* < 0.0001 at the following time points) significantly prolonged PWT compared to the MIA + vehicle group at different time points (Fig. 4a).

Similar findings were obtained on PWL (*p* < 0.0001 at all time points for any treatment), with the only difference that the effect of the lower dose of PEA-Q became significant at the seventh day (*p* < 0.0001 at 7, 14 and 21 days post-injection, Fig. 4b). The sham group showed no evident time-dependent changes (Fig. 4a, b). PEA-Q at 20 mg/kg dose resulted to exert a superior effect on both PWT and PWL compared to the lower dose and to meloxicam (*p* < 0.0001 for both variables and both comparisons, at any time point) (Fig. 4a, b). Furthermore, rats treated with PEA-Q at 20 mg/kg exhibited nociceptive behaviors that were not statistically different from the sham group up to day 14 and 21 for PWT and PWL respectively. In addition, motor function at different time points was assessed by walking track analysis. The sham group showed normal motor activity, with SFI approximating 0. In the MIA + vehicle group, SFI values were significantly lower than in sham animals, locomotor function being increasingly impaired at 3, 7, 14 and 21 days (Fig. 4c) (*p* < 0.0001 at all time points). Treatment with both PEA-Q and meloxicam significantly improved locomotor function at both 14 and 21 days (*p* < 0.0001 for each treatment and either time points) with no statistically significant differences being observed among treatments (Fig. 4c).

#### B. Effect of PEA-Q on MIA-induced histopathological changes

**Fig. 5:**
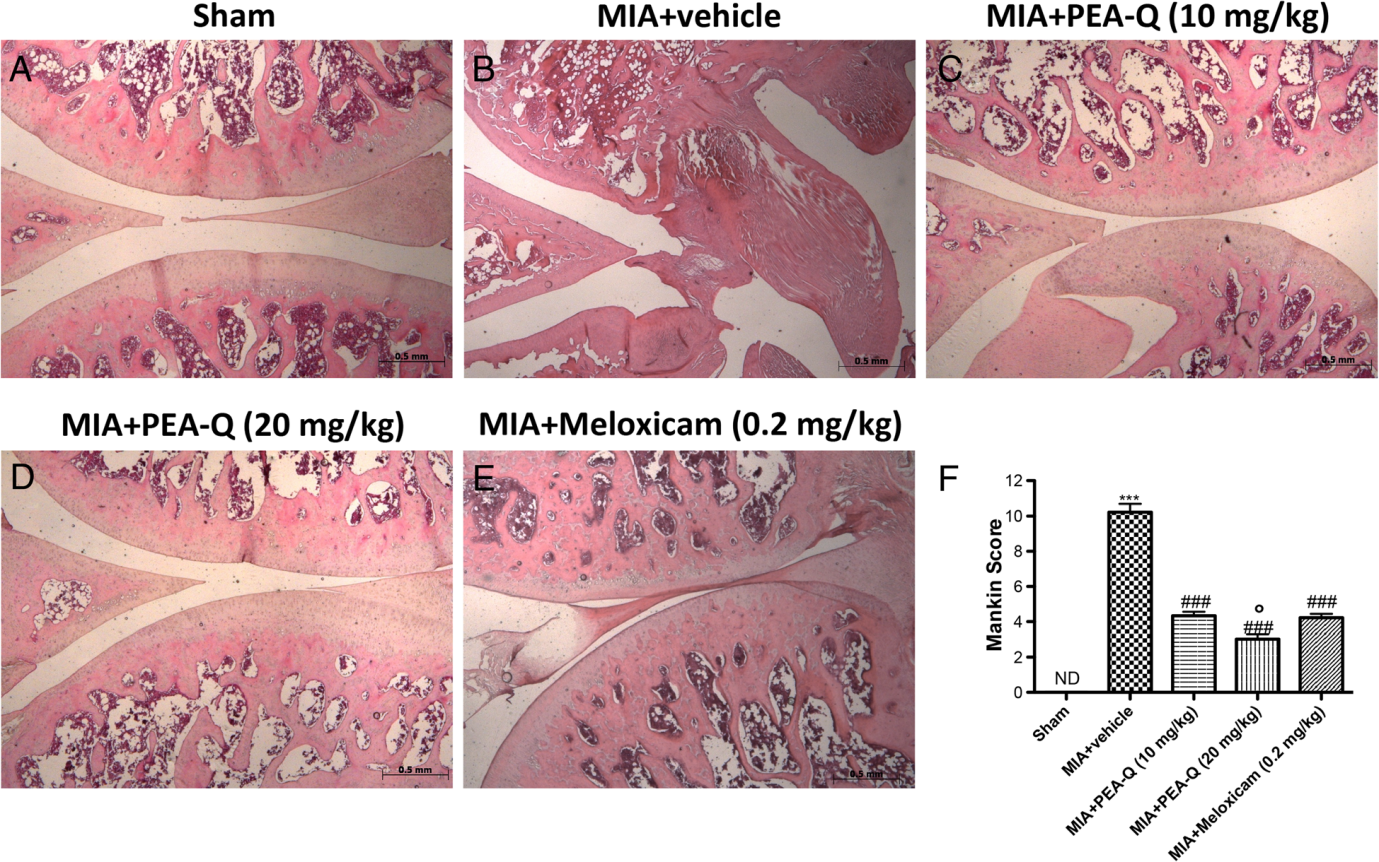
Effects of PEA-Q on MIA-induced histological features of OA knee tissue. Histological evaluation was performed by haematoxylin and eosin staining. Panel (**a**), sham; panel (**b**), MIA–injected; Panels (**c**) and (**d**), PEA-Q treatment; Panels **d** and (**e**), meloxicam treatment. Figures are representative of all animals in each group. Panel (**f**), Mankin score for the various treatment groups. Values are means ± SEM of 10 animals for each group. ***p < 0.0001 vs sham. ^###^ *p* < 0.0001 vs MIA + vehicle. °*p* < 0.05 vs MIA + PEA-Q (10 mg/kg) and MIA + meloxicam

Histological examination of haematoxylin/eosin-stained knee sections 21 days after intra-articular injection of MIA showed a reduction in blood cells, irregularities in the surface layer, and multi-layering in transition and radial zones of the cartilage compared to sham (Fig. 5a, b see Mankin score F) (*p* < 0.0001). Treatment with PEA-Q at both doses reduced the histological cartilage changes induced by MIA injection (Fig. 5c,d see Mankin score F) (*p* < 0.0001 for either doses), the higher dose showing superior effect compared to the lower dose (*p* = 0.0058) and meloxicam (*p* = 0.0113, Fig. 5e see Mankin score F).

#### C. Effects of PEA-Q on plasma concentration of inflammatory, nociceptive and matrix degradation markers

**Fig. 6:**
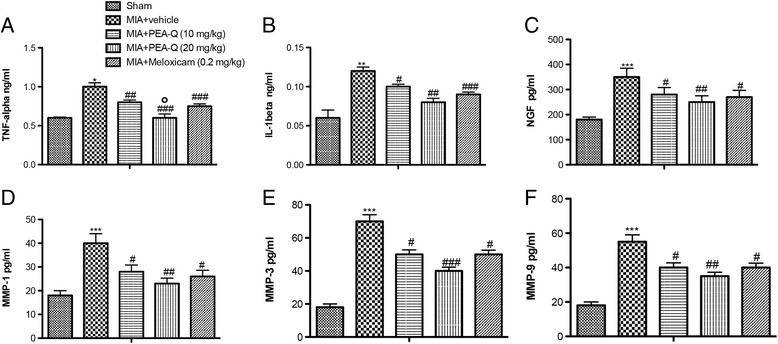
Effects of PEA-Q on MIA-induced plasma cytokines, metalloproteinases and NGF in OA rats. Increased TNF-α (**a**) IL-1β (**b**), NGF (**c**) and MMP1 (**d**), MMP-3 (**e**), MMP-9 (**f**) plasma concentrations were detected in the MIA + vehicle group compared to sham animals. PEA-Q treatment significantly reduced the plasma concentration of all measured parameters, with effects being comparable to the meloxicam group. Values are means ± SEM of 10 animals for each group. *p < 0.05, **p < 0.001 and***p < 0.0001 vs sham. ^#^ *p* < 0.05, ^##^ *p* < 0.001 and ^###^ *p* < 0.0001 vs MIA + vehicle. °*p* < 0.05 vs MIA + PEA-Q (10 mg/kg) and MIA + meloxicam. The exact *p values* are reported in the text

A significant increase in serum concentration of the pro-inflammatory cytokines TNF-α (*p* = 0.0047, Fig. 6a) and IL-1β (*p* = 0.0007, Fig. 6b) and a similar increase in the level of the nerve sensitizer NGF (*p* < 0.0001, Fig. 6c) were observed in the MIA + vehicle group compared to sham animals. Treatment with PEA-Q significantly counteracted such increases at both 10 mg/kg (TNF-α, *p* = 0.0003; IL-1β, *p* = 0.0029; NGF, *p* = 0.0226) and 20 mg/kg dose (TNF-α, *p* < 0.0001; IL-1β, *p* = 0.0001; NGF, *p* = 0.0002). Similar findings were observed for meloxicam (TNF-α, *p* < 0.0001; IL-1β, *p* < 0.0001; NGF, *p* = 0.0017). Limited to TNF-α concentration, PEA-Q at the higher dose sowed a superior effect compared to the lower dose (*p* = 0.0070) and to meloxicam (*p* = 0.0443).

In addition, MIA + vehicle rats showed a markedly higher expression of the matrix degradation enzymes MMP-1 (Fig. 6d), MMP-3 (Fig. 6e), and MMP-9 (Fig. 6f) compared to the sham group (*p* < 0.0001 for all the three). PEA-Q significantly decreased MMP concentration compared to the vehicle-treated group, both at the 10 mg/kg dose (MMP-1, *p* = 0.0120; MMP-3, *p* = 0.0032; MMP-9, *p* = 0.0142) and at the 20 mg/kg one (MMP-1, *p* = 0.0002; MMP-3, *p* < 0.0001; MMP-9, *p* = 0.0003), similar to that observed with meloxicam (MMP-1, *p* = 0.0019; MMP-3, *p* = 0.0011; MMP-9, *p* = 0.0044) (Fig. 6d–f). No statistically significant difference was observed between different PEA-Q doses or between either dose of PEA-Q and meloxicam.

## Discussion

Here we show for the first time that a new co-ultramicronized composite, made of the anandamide congener PEA and the natural polyphenol quercetin (PEA-Q), exerts beneficial effects in both inflammatory and mixed persistent OA pain in rats. Although the anti-inflammatory and anti-hyperalgesic activities of PEA have been clearly demonstrated in several inflammatory and neuropathic pain models [13–15, 31], reports on joint disease-related pain states are scarce, with no study having yet been performed with PEA-Q. Conceivably, PEA-Q could dissociate into its single components (PEA and quercetin) after oral administration, although there is evidence supporting the synergistic effect of PEA and polyphenols [32]. OA pain is, without doubt, complex in its cellular/molecular mechanisms. As such, designing therapeutics that provide optimal efficacy may call for the use of models that allow one to evaluate distinct features of the disease in the context of the agent being evaluated. Indeed, we used both the CAR paw oedema and MIA-induced OA models, since previous studies gave information about the potential effect of the two components of PEA-Q on both inflammatory and neuropathic pain.

Intraperitoneal administration of ultramicronized PEA, alone or co-micronized with the polyphenol luteolin, is able to restore normal pain sensitivity and decrease inflammation in a rat model of rheumatoid arthritis [32]. In human patients, a two-week oral treatment with micronized PEA showed superior activity compared to the NSAID ibuprofen in decreasing temporomandibular joint inflammatory pain and improving joint function (better mandibular range of motion) [33]. Further, pharmacological inhibition of PEA degradation (which elevates tissue PEA levels), results in anti-hyperalgesic effects in arthritic pain models [34–36]. The pain relieving activity observed for PEA-Q is in line with these earlier findings, although direct comparisons are difficult due to different models and PEA formulations employed.

Quercetin has been reported to exert pain-relieving effects in different pain models [37] and, in particular, is able to reduce: (i) CAR-induced mechanical hyperalgesia [37], (ii) chemotherapy-induced neuropathic pain [38], (iii) diabetic neuropathic pain [37], and (iv) muscle mechanical hyperalgesia [39]. We were unable to confirm these observations, since quercetin did not show any significant effect on paw oedema or thermal sensitivity in the CAR-induced inflammatory pain model. This apparent lack of effect could result from differences in dosage used: we tested quercetin at 3.3 mg/kg (equivalent to 20 mg/kg PEA-Q), while the dosage used in the above-mentioned studies was approximately 100 mg/kg. Further synergistic studies will be needed to better characterize the contribution of quercetin to the activity of PEA-Q.

It was somewhat surprising that meloxicam exerted an anti-allodynic effect in the MIA model (significant improvement of PWL and PWT). NSAIDs are usually considered to be ineffective on allodynia, as recently shown in naturally-occurring OA in cats subjected to punctuate tactile allodynia quantification [40]. Again, differences in dosages used in the former study and ours, as well as species differences and nature of the disease (spontaneous versus chemically-induced OA) could be responsible for the apparent discrepancy. Although allodynia might not be the main target of meloxicam, its ability to improve functional parameters and pain scores in dogs with joint pain has been repeatedly demonstrated [41, 42]. Our findings on improved mechanical sensitivity and motor function following meloxicam are in line with the above-cited studies. PEA-Q also significantly improved locomotor function, as measured by walking track analysis. Interestingly, this analysis combines gait with the temporal and spatial relationship of one footprint to another during walking [29]. MIA injection is known to cause dropping of the foot to the ground, resulting in visible footprint changes [29]. One can envisage that these changes are associated with enhanced nociception and “antalgic gait”. That is to say, OA animals minimize contact with the floor during walking. This could well correlate with the situation of OA dogs, in which walking likely causes enhanced nociception in the affected limb(s) leading to lameness. The decrease of walking alterations observed in our study might thus be clinically relevant and can be considered a measure of the motor functional recovery exerted by PEA-Q, as well as meloxicam.

The ability to decrease not only pain and locomotor deficits, but also inflammation is a further point in favor of the therapeutic use of PEA-Q in animals affected with OA. Inflammation and related soluble mediators are involved in: (i) the generation of acute pain, (ii) the vicious cycle of pain maintenance and chronicity, and (iii) the predominance of catabolic processes over anabolic ones, ultimately resulting in tissue degradation and damage at the cartilage and bone levels [7]. PEA-Q significantly reduced inflammatory paw oedema, and is in accordance with data obtained in the same model using either PEA [23, 43, 44], or PEA co-ultramicronized with various polyphenols [45]. Interestingly, PEA-Q showed a longer anti-inflammatory effect compared to meloxicam, whose effect lost significance at an earlier time point.

Neither meloxicam nor quercetin achieved statistical significance in decreasing histological signs of inflammation, while oral treatment with PEA-Q resulted in a clear reduction in histological inflammatory score. The effect was paralleled by a significant reduction in MPO activity (the most abundant pro-inflammatory enzyme of neutrophilic granulocytes) in PEA-Q treated rats, which proved to be superior to meloxicam in reducing neutrophil infiltration in paw tissues. These results are in agreement with previous studies, showing that PEA decreased inflammatory cell infiltration in different inflammatory models [23, 46, 47]. Notably, the increase of pro-inflammatory, nociceptive and proteolytic markers analyzed was significantly counteracted by PEA-Q to the same extent (and even better concerning TNF-α) as with meloxicam. Interestingly, cytokines (e.g., IL-1β, TNF-α) and MMPs are considered to play a crucial role in chondrocyte cell death and matrix degeneration [7, 48] and NGF is currently viewed as a key regulator of nociceptive and neuropathic pain [49]. Given that (i) the concentration of NGF in canine synovial fluid is significantly increased in chronically lame dogs [50], and (ii) feline OA-associated pain improves under treatment with a anti-NGF antibody [51], the ability of PEA-Q to reduce NGF levels during experimentally-induced OA holds promise for a disease-modifying effect against pain in canine and feline OA patients.

PEA-Q counteracted histological OA changes caused by MIA injection in the tibiofemoral joint and significantly decreased the severity of cartilage degeneration, the effect being superior to meloxicam. To the best of our knowledge no study has investigated the effect of either PEA or quercetin on histological joint damage, and so it is difficult to compare our data with previous findings. The only available data in this regard involves the effectiveness of intra-articular injected meloxicam, which decreased joint histologic score in MIA rats - albeit without reaching statistical significance [52].

Although complete extrapolation of efficacy from the pre-clinical models used here to veterinary patients would be unadvised, the present findings shed new light on some of the inflammatory and nociceptive pathways and molecules targeted by PEA-Q. In the present study, the use of two different models and assessment of the effect at different levels (behavioural, tissue, and molecular) to a large extent address the most relevant questions. Moreover, the pain-relieving effect of PEA-Q not only on CAR-inflammation but also on MIA-related pain supports its use in OA pain states, where inflammatory and neuropathic mechanisms frequently coexist. Studies on the effects of PEA-Q on pain and locomotion in lame dogs are in progress.

## Conclusions

PEA-Q is a novel co-ultramicronized formulation of PEA and quercetin whose effects were investigated in two pre-clinical models of OA pain in rats. Oral administration of PEA-Q decreased pain sensitivity, improved locomotor function, reduced inflammatory signs and mediators and lowered histological damage score. Although the underlying mechanism(s) of the observed effects are beyond the scope of our study, the particular cellular targets of PEA (e.g., mast cells and microglia) [19], redundancy of its receptors (direct and direct agonism on nuclear and membrane cannabinoid receptors) [14] and oxidative stress addressed by quercetin [22, 37] comprise targets that could be different from standard pharmacological tools (i.e., NSAIDs). Importantly, toxicological studies show that micronized and ultramicronized PEA is safe, the LD50 being greater than 2000 mg/kg [53]. Individually or in association with different antioxidant polyphenols, micronized and ultramicronized PEA has a long track record of use in human and veterinary patients, with good-to-excellent tolerability [14]. Moreover, prolonged use of PEA is not associated with the development of tolerance [43, 54]. There is an unmet need in veterinary medicine for the development of new agents to treat OA-associated pain which target alternative mechanisms distinct from currently approved drugs. The collective observations presented here propose that PEA-Q shows promise for multimodal pain management in canine and feline OA.
